# Hydrogen sulfide prevents arterial medial calcification in rats with diabetic nephropathy

**DOI:** 10.1186/s12872-021-02307-9

**Published:** 2021-10-13

**Authors:** Fang-Zheng Wang, Hong Zhou, Hong-Yu Wang, Hang-Bing Dai, Qing Gao, Pei Qian, Ye-Bo Zhou

**Affiliations:** Department of Physiology, Nanjing Medical University, 101 Longmian Road, Nanjing, 211166 Jiangsu China

**Keywords:** Arterial medial calcification, Hydrogen sulfide, Diabetic nephropathy, Elastin, Stat3, Cathepsin S

## Abstract

**Background:**

Arterial medial calcification (AMC) is associated with a high incidence of cardiovascular risk in patients with type 2 diabetes and chronic kidney disease. Here, we tested whether hydrogen sulfide (H_2_S) can prevent AMC in rats with diabetic nephropathy (DN).

**Methods:**

DN was induced by a single injection of streptozotocin and high-fat diet (45% kcal as fat) containing 0.75% adenine in Sprague–Dawley rats for 8 weeks.

**Results:**

Rats with DN displayed obvious calcification in aorta, and this was significantly alleviated by Sodium Hydrosulfide (NaHS, a H_2_S donor, 50 μmol/kg/day for 8 weeks) treatment through decreasing calcium and phosphorus content, ALP activity and calcium deposition in aorta. Interestingly, the main endogenous H_2_S generating enzyme activity and protein expression of cystathionine-γ-lyase (CSE) were largely reduced in the arterial wall of DN rats. Exogenous NaHS treatment restored CSE activity and its expression, inhibited aortic osteogenic transformation by upregulating phenotypic markers of smooth muscle cells SMα-actin and SM22α, and downregulating core binding factor α-1 (Cbfα-1, a key factor for bone formation), protein expressions in rats with DN when compared to the control group. NaHS administration also significantly reduced Stat3 activation, cathepsin S (CAS) activity and TGF-β1 protein level, and improved aortic elastin expression.

**Conclusions:**

H_2_S may have a clinical significance for treating AMC in people with DN by reducing Stat3 activation, CAS activity, TGF-β1 level and increasing local elastin level.

**Supplementary Information:**

The online version contains supplementary material available at 10.1186/s12872-021-02307-9.

## Introduction

Arterial medial calcification (AMC) promotes the cardiovascular morbidity and mortality in patients with diabetes mellitus (DM) or chronic kidney disease (CKD) [1, 2]. It is an active and regulative process that is similar to osteogenesis [3, 4]. Metabolic disorders such as hyperglycemia and hyperphosphatemia existing diabetes (e.g. elevated glucose level) and uremia (e.g. elevated phosphorus level) [3, 4] could aggravate AMC. Vascular calcification (VC) is often found in patients with CKD, particularly in those with DM [5, 6], which can further contribute to the substantial increase in cardiovascular event [7–9].

Osteogenic transition of vascular smooth muscle cells (VSMCs) is closely related to AMC [10, 11], which is accompanied by increases of calcium and phosphorus contents, alkaline phosphatase (ALP) activity and core binding factor α-1 (Cbfα-1) protein expression, an important transcription factor that regulates osteogenic differentiation, and decreases of protein expressions of SM α-actin and SM22α, two phenotypic markers highly expressed in VSMCs [1, 12]. High level of glucose can induce osteogenic transition of VSMCs in the presence of high concentration of phosphorus [3, 13].

Hydrogen sulfide (H_2_S) has been widely explored in the animal models with cardiovascular and kidney diseases [14–16]. Cystathionine-γ-lyase (CSE) is mainly responsible for H_2_S production in arterial wall [17], and CSE/H_2_S system is important for the maintenance of VSMCs differentiation. However, reduced CSE activity is involved with cardiovascular diseases such as hypertension and myocardial dysfunction in diabetes [18]. In hyperglycemic state, H_2_S can suppress VSMCs proliferation [19]. It also decreases blood glucose, and has an effective role in prevention of cardiovascular diseases such as atherosclerosis and diabetic cardiomyopathy in diabetic rats [20]. Moreover, H_2_S significantly involves the renal protection [16, 21, 22]. More interestingly, H_2_S treatment showed obvious improvement in biochemical abnormalities in rats with diabetic nephropathy (DN) [22]. Therefore, it can be speculated that H_2_S may have a beneficial role in the treatment of AMC in rats with DN. More importantly, some studies have found that H_2_S can attenuate VC and suppress osteogenic transformation of VSMCs [13, 23]. Especially, it has been confirmed that H_2_S attenuates VSMCs calcification induced by high levels of glucose and phosphate through upregulating elastin level via the inhibition of Stat3/Cathepsin S (CAS) signaling [13]. But it is unclear whether H_2_S can prevent AMC by means of similar mechanisms in animal model with diabetic nephropathy (DN, e.g. accompanied by high glucose and phosphorus levels).

As one of signal transducer and activator of transcription (Stat) family members, Stat3 can promote signal integration in the vascular dysfunction [24–26]. Cathepsin S (CAS) cleaves elastin and generates bioactive elastin peptides, which contribute to calcification [27, 28]. Moreover, Stat3 and CAS are closely associated with the occurrence and development of calcification of VC [29, 30]. Especially important, Stat3-mediated activation of CAS involved in calcification of VSMCs has been confirmed under the stimulation of high levels of glucose and phosphorus [13]. STAT3 also enhances hepatic fibrosis through the upregulation of TGF-β1 expression [31], which contributes to the calcification of VSMCs involving the mechanisms of TGF-β1 pathway [32]. It has been previously shown that elastin degradation products work synergistically with TGF-β1 to induce osteogenesis in vascular smooth muscle cells [33].

Therefore, this study was designed to investigate the protective effects of H_2_S on AMC in rats with DN and its possible mechanisms.

## Materials and methods

### Reagents

Glycine, Tris, SDS, mannitol, NaCl, bovine serum albumin (BSA), STZ and sodium hydrosulfide (NaHS) were purchased from Sigma-Aldrich (St Louis, MO. USA). Bradford colorimetric protein assay kit (Rockford, IL.USA) was used for protein quantification. The RIPA buffer was from Thermo Fisher Scientific Inc (Waltham, MA. USA). The phosphorus, calcium and alkaline phosphatase (ALP) kits were purchased from Jiancheng Bioengineering Co (Nanjing, China). High fat diet with 0.75% adenine was from TROPHIC Animal Feed High-tech Co Ltd (Nantong, China). Other chemicals and reagents were of analytical grade.

### Rat DN model

Animal protocols were approved by the Experimental Animal Care and Use Committee of Nanjing Medical University, and we complied with the Guide for the Care and Use of Laboratory Animals (NIH publication, 8th edition, 2011). Male Sprague–Dawley rats weighing 200–250 g rats were from Animal Laboratory Center of Nanjing Medical University. DN model (16 rats) was created with a high-fat diet (45% kcal as fat) containing 0.75% adenine, and a single intraperitoneal injection of STZ (ip, 35 mg/kg; Sigma) dissolved in sodium citrate buffer (pH 4.5). Control rats (n = 14) were only injected with sodium citrate buffer (pH 4.5). NaHS (50 μmol/kg/day) was administered intraperitoneally to rats after a single injection of STZ (n = 8) or control rats (n = 7). NaHS and high-fat diet containing 0.75% adenine were started 1 week (7 days) after injection of STZ and were maintained for 8 weeks. Control rats (n = 7) were fed with normal diet (12% kcal as fat) and injected saline for 8 weeks. At the end of the study, animals were euthanized, and plasma and tissues were collected.

### Calcification determination

The aortic specimens were fixed in 4% formaldehyde in phosphate-buffered saline and were paraffin embedded. Specimens were cut into 6 μm sections and underwent haematoxylin and eosin (HE) and alizarin red staining (positive calcium staining is orange/red). Sections were fixed with 4% formaldehyde for 10 ~ 15 min and washed out, followed by an incubation with alizarin red (1%, wt/vol, pH 4.2) for 5 min, and rinsed by distilled water for half an hour or so.

### Measurement of H_2_S levels in plasma

H_2_S concentrations were detected spectrophotometrically in plasma by a colorimetric assay. Briefly, 50 µL distilled water were mixed with 100 µL plasma samples in tubes containing 300 µL zinc acetate (1%). 200 µL *N*,*N*-2-dimethyl-*p*-phenylenediamine sulfate (20 mM in 7.2 M HCl) was added to terminate the reaction and followed by 200 µL FeCl3 (30 mM in 1.2 M HCl) addition. 150 µL trichloroacetic acid (10%) was added to precipitate protein from the samples. The supernatant absorbance was measured at 665 nm by a microplate reader (Thermo Electron Corporation. Waltham, MA. USA). H_2_S concentrations were assessed using a curve of standard H_2_S solutions (NaHS: 3.125–100 µM).

### Calcium content measurement

The calcium content in the plasma or aortic media was determined by using O-cresolphthalein colorimetric (OCPC) method. HCl was used for aorta decalcification. The supernatant fluid was added into the mixed working reagent solution containing ethanolamine buffer, 8-hydroxyquinoline and OCPC, and incubated at 30 °C for 5 min. The absorbance of this final solution was measured by a microplate reader (Thermo Electron Corporation. Waltham, MA. USA) at 600 nm [13].

### Phosphorus level measurement

Phosphorus content in the plasma and aortic media was determined by phosphomolybdic acid method. The plasma or aortic tissue homogenate were mixed by precipitating agent. Then the mixed solution was centrifuged, and the supernatant fluid was added into the working solution containing hosphomolybdic acid. The final solution was incubated, and the absorbance was determined by a microplate reader (Thermo Electron Corporation. Waltham, MA. USA) at 660 nm [13].

### Alkaline phosphatase (ALP) measurement

The proteins homogenate of aorta was made in 0.05% Triton X-100 in PBS. Total bicinchoninic acid (BCA) protein assay was used to quantify total proteins. The supernatant samples were mixed with reaction mixture and incubated at 37 °C for 15 min, then the absorbance was determined at 520 nm after the developer addition. ALP activity was calculated according to one unit was defined as 1 g tissue protein producing l mg phenol for 15 min. Results were normalized to levels of total protein.

### Cystathionine-γ-lyase (CSE) activity detection

H_2_S synthesis enzyme CSE activity in aorta was measured with the H_2_S production rate. The same amount of aortic tissue of each rat was homogenized with 100 mM potassium phosphate buffer (pH 7.4). 10 mM l-cysteine and 2 mM pyridoxal 5’-phosphate were also added into the buffer, then the mixed solution was incubated at 37 °C for 30 min. H_2_S was captured by the zinc acetate (1% wt/vol) addition, and trichloroacetic acid (10% wt/vol) was used to stop the reaction. Finally, equal volumes of FeCl_3_ (30 mM) and *N*,*N*-dimethyl-*p*-phenylenediamine sulfate (20 mM) were added, and the final solution was centrifuged for 10 min. The supernatant absorbance was measured at 670 nm [34].

### CAS activity measurement

After protein quantification, the homogenate of aorta (100 µg) was incubated in chilled buffer for 10 min and then centrifuged for 5 min. 2 µL of the 10 mM substrate was transferred into the 96-well plate, and 50 µL of reaction buffer and 50 µL of supernatant were added orderly into the plate. The mixed solution was incubated at 37 °C for 2 h. At the wave length of 400 nm excitation and 505 nm emission, the samples were detected by a microplate reader (Thermo Electron Corporation. Waltham, MA. USA).

### Measurement of plasma glucose, creatinine and urea nitrogen levels

At the end of 8 weeks of NaHS treatment, all rats fasted overnight for 12 h without diet but had free access to water before experiments. The next morning, about 1.0 mL of blood was collected from tail vein at around 8 o’clock for the measurement of fasting plasma glucose level through glucose oxidase method by using a kit from Jiancheng Bioengineering (Nanjing, China). After collecting blood from the tail vein, an overdose of sodium pentobarbital was used to anaesthetize each rat by intraperitoneal injection. At first, the rats were anesthetized with the normal dose according to the weight (0.3 mL/100 g). If the animals were not completely anesthetized, then we increased the dose about a fifth of the total amount. At this dose, most animals can be completely anesthetized. For better anesthetic effect, 2% sodium pentobarbital solution was prepared before operation. Plasma samples were obtained by centrifugation of heparinized blood for estimation of circulating creatinine and urea nitrogen levels by using commercial colorimetric assay according to the manufacturer’s instructions. The kits were from Jiancheng Bioengineering (Nanjing, China). At the same time, the aortic tissue was quickly removed. One part was fixed in 4% formaldehyde in phosphate-buffered saline and the other part was frozen with liquid nitrogen. Finally, the plasma and the frozen aortic tissue were stored at − 80 °C until being used.

### Western blot analysis

Total aortic protein in the homogenate was extracted and measured. The protein expressions of SM22α, SMα-actin, elastin, Cbfα-1, CSE, total-Stat3, phosphorylated-Stat3, CAS and TGF-β1 in the aorta were determined by Western blot analysis [13]. Briefly, the proteins on nitrocellulose membrane were probed with primary antibodies against SM22α, SMα-actin, elastin, CSE (Santa Cruz Biotechnology, Santa Cruz, Calif., USA), total-Stat3, phosphorylated-Stat3 and Cbfα-1 (Cell Signaling Technology, 3 Trask Lane Danvers, MA.,USA), CAS (Abcam, Cambridge, Mass., USA) or TGF-β1 (Affinity Bioscience, Cincinnati, OH., USA). Horseradish peroxidase-conjugated anti-mouse, anti-rabbit or anti-sheep IgG were used as secondary antibody. The immunodetection was performed using autoradiography. The levels of protein band intensities were normalized with β-actin Stat3 or non-phosphorylated Stat3 levels. The original and unclipped Western blotting images corresponding to clipped Western blotting images in the manuscript were shown in Additional file 1.

### Statistical analysis

Data are expressed as the mean ± S.E. Comparisons between two observations were assessed by the unpaired Student’s t test. One-way ANOVA followed by Bonferroni’s post-hoc test was used analyze multiple comparisons. *p* < 0.05 was considered statistically significant.

## Results

### NaHS treatment attenuates the development of DN in rats

**Fig. 1:**
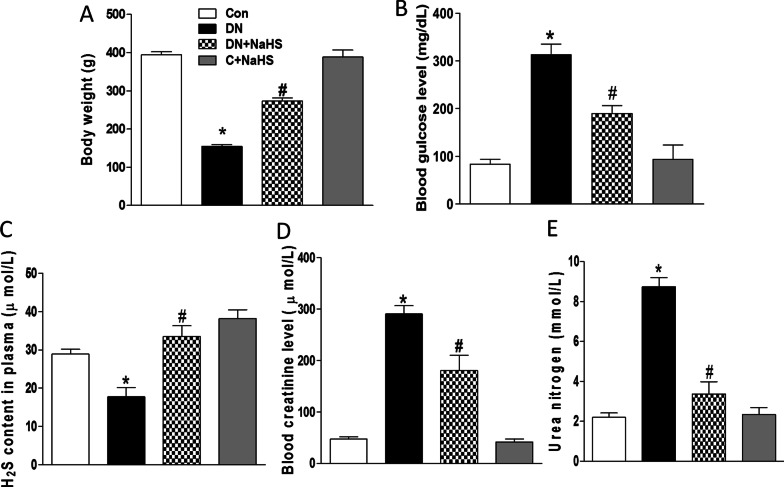
Generation of rats with diabetic nephropathy (DN).The rats were fed a high fat diet containing 0.75% adenine for 8 weeks and performed a single STZ (35 mg/kg) injection intraperitoneally. Rats were divided into four groups: control rats fed with normal diet and saline injection (**C**), high fat and adenine-fed and STZ-injected rats (DN), DN rats with sodium hydrosulfide (NaHS) treatment (DN + NaHS), and control rats with NaHS treatment (C + NaHS), **A**–**E**, Body weight (**A**), the concentration of glucose (**B**), H_2_S (**C**), creatinine (**D**), and urea nitrogen (**E**) in plasma were determined. n = 6 ~ 7 rats. Values represent the means ± SEM.*p < 0.05 compared to control rats. #*p* < 0.05 compared to DN rats

A significant decrease in body weight (~ 155 g) (Fig. 1A) and marked increased blood levels of glucose (Fig. 1B), creatinine (Fig. 1D) and urea nitrogen (Fig. 1E) were found in the rats treated with one injection of STZ, and high fat diets and adenine for 8 weeks (DN rats) when compared with those in the control group, indicating that a successful DN rat model was created. The above changes were significantly improved by the treatment of NaHS (50 μmol/kg/day, ip, 8 weeks). These data suggest that H_2_S may be beneficial to treat DN.

### NaHS treatment attenuates calcification in aortic tissue of DN rats

**Fig. 2:**
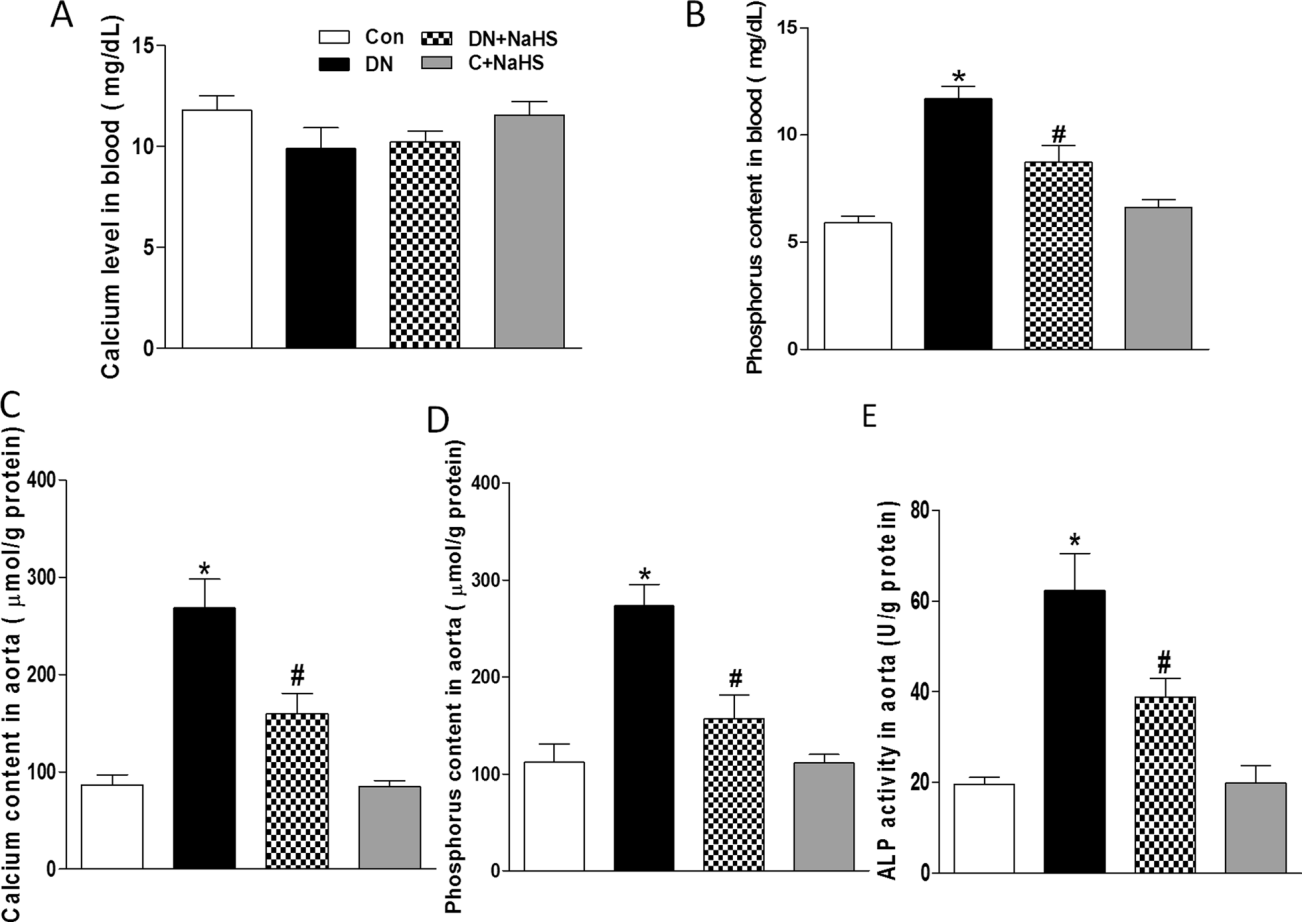
Formation of arterial medial calcification in rats with DN. Plasma concentrations of calcium (**A**), phosphorus (**B**), aortic calcium (**C**), phosphorus (**D**) contents, and ALP activity (**E**) were detected after 8 weeks of NaHS (50 μmol/kg/day) treatment. ALP: alkaline phosphatase. n = 6 ~ 7 rats. Values represent the means ± SEM. *p < 0.05 compared to control rats. #*p* < 0.05 compared to DN rats

VC was evaluated by Ca deposition, calcium and phosphorus content, and ALP activity in aorta. Compared to the Control group, the marked increased blood level of phosphorus (Fig. 2B) was found, but not calcium levels (Fig. 2A) in DN rats, and there were also significant increases in levels of calcium (Fig. 2C) and phosphorus (Fig. 2D), and ALP activity (Fig. 2E) in the aorta of DN rats, which were significantly attenuated by NaHS treatment. It confirms that H_2_S is able to attenuate the aortic calcification in rats with DN.

### NaHS treatment improves aortic remodeling and reduces calcium deposition in tunica media of aorta of DN rats

**Fig. 3:**
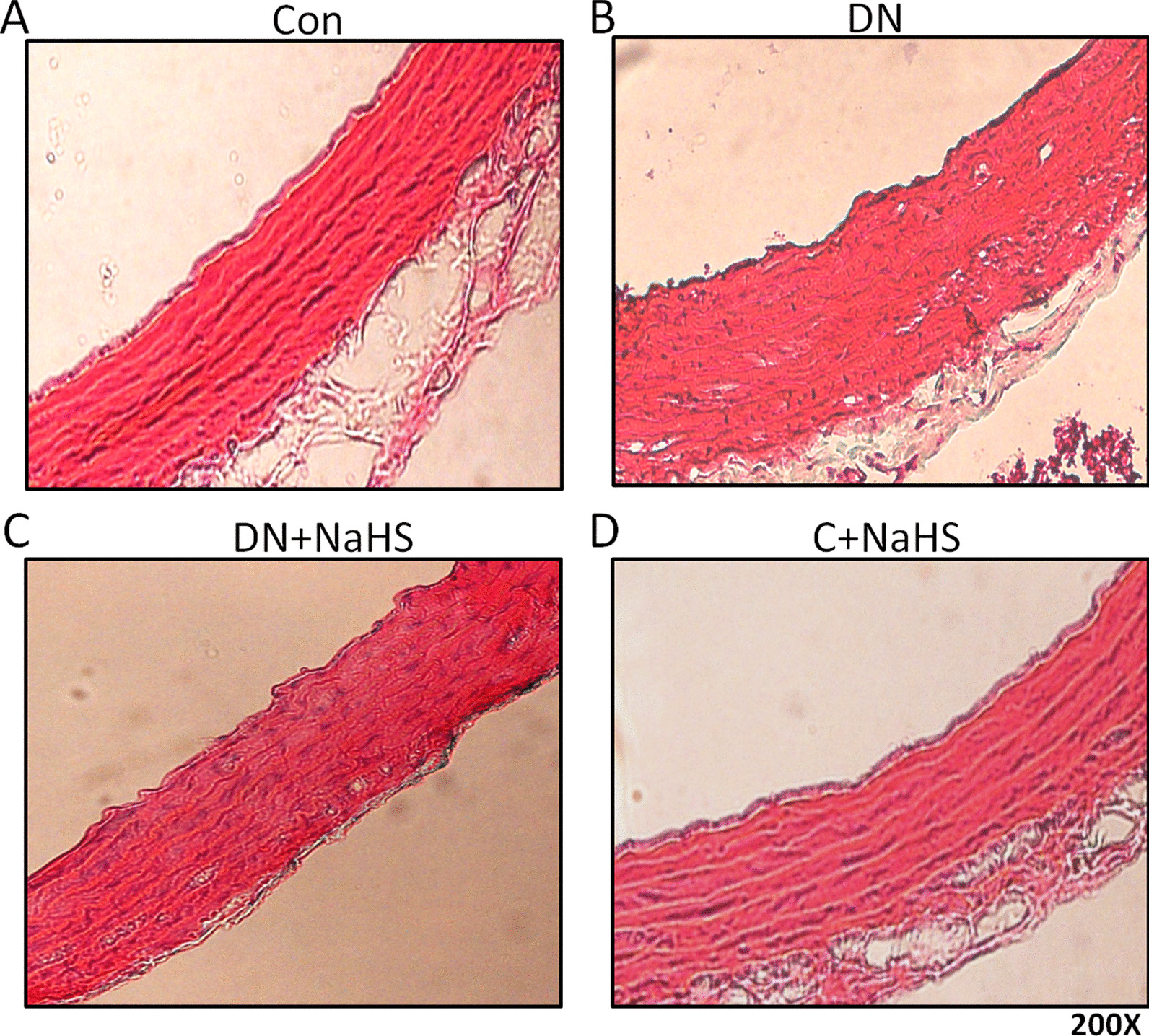
NaHS improved arterial vascular remodeling. Aortas underwent haematoxylin and eosin (H&E staining, magnification 200 ×). n = 3 ~ 4 rats

**Fig. 4:**
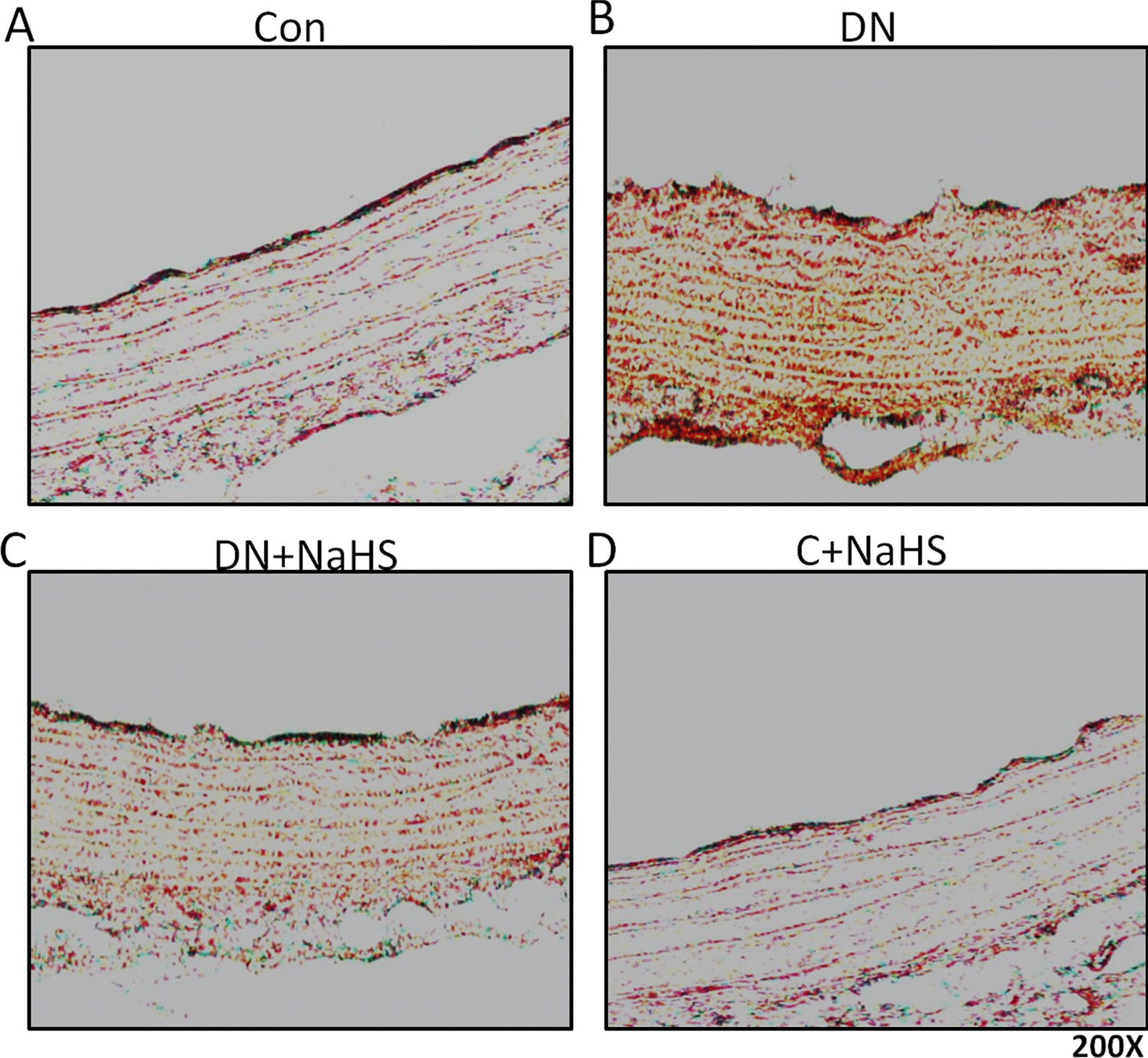
NaHS prevented arterial medial calcification. Aortas underwent alizarin red staining (magnification 200 ×). n = 3 ~ 4 rats

When compared to the Control group (Figs. 3A, 4A), HE and Alizarin red staining showed irregular elastic fibers (Fig. 3B, HE staining) and Ca deposition (Fig. 4B, Alizarin red staining) in tunica media of aorta of DN rats, which were significantly improved by NaHS treatment (Figs. 3C, 4C). However, NaHS treatment had no obvious changes in aortic remodeling and calcium deposition in normal rats when compared to the Control group (Figs. 3D, 4D).

### Decreased plasma H_2_S levels and impaired endogenous H_2_S generating enzyme cystathionine-γ-lyase (CSE) activity and expression in DN aorta, and NaHS treatment inhibits osteogenic transition of VSMC in aorta of DN rats

**Fig. 5:**
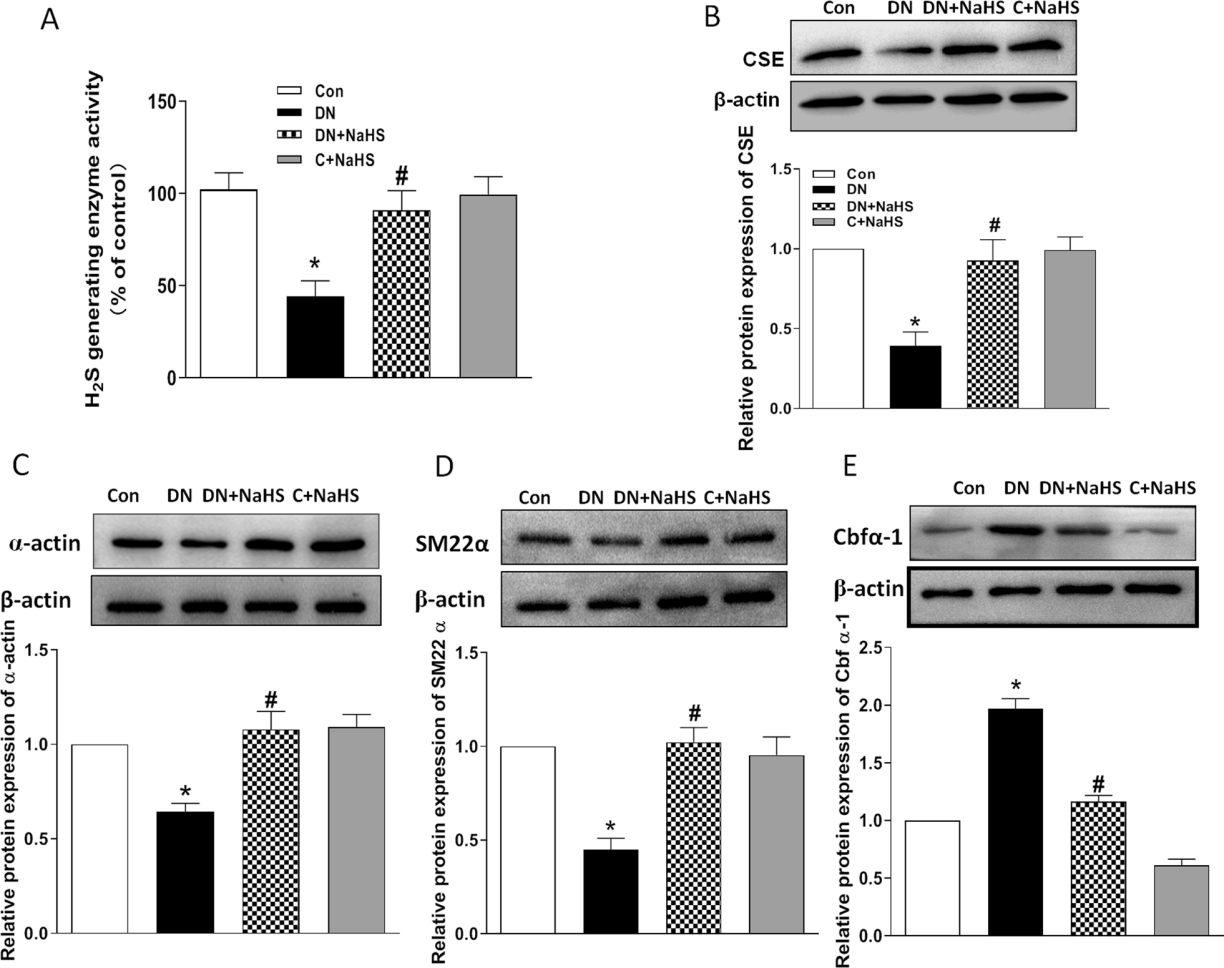
NaHS restored the H_2_S generating enzyme activity and its protein expression (**A**, **B**), and depressed osteogenic transition in medial smooth muscle cells (SMC) of aorta in DN rats (**C**, **D**, **E**). Western blot method was used to examine the protein expressions of α-actin (**C**), SM22α (**D**) and Cbfα-1 (**E**). β-actin from the same blot was control for protein loading. α-actin and SM-22α are smooth-muscle lineage markers, and core binding factorα-1 (Cbfα-1) is osteogenic marker. Results are from one representative experiment and relative to β-actin. CSE: cystathionine-lyase. C: control. n = 6 ~ 8 rats (**A**) and n = 3 ~ 5 rats (**B**, **C**, **D**). Values represent the means ± SEM. *p < 0.05 compared to control rats. #*p* < 0.05 compared to DN rats

We explored the ability of H_2_S generating enzyme CSE for demonstrating the role of endogenous H_2_S. The results showed that H_2_S levels in plasma (Fig. 1C) and CSE activity in aora were largely decreased in DN rats (Fig. 5A). Since CSE is the main enzyme to produce H_2_S in vascular vessels, we further measured CSE protein expression in aortic tissue. Western blots demonstrated that CSE level was also significantly reduced in aorta (Fig. 5B). However, the decreased CSE activity and protein expression were improved by NaHS treatment in DN rats. These data indicate that endogenous H_2_S is decreased in plasma during diabetic nephropathy and the impaired endogenous H_2_S generating enzyme ability in aorta may contribute to the vascular abnormalities of structure and function in DN. SM22α and α-actin, two phenotypic markers of VSMC, and Cbfα-1, a key osteogenic regulator, in tunic media were used to assess the osteogenic transition. We examined their protein expressions in aorta. As shown in Fig. 5, the protein expressions of α-actin (Fig. 5C) and SM22α (Fig. 5D) were decreased, whereas the protein expression of Cbfα-1 (Fig. 5E) was increased in the aortic tissue of DN rats. However, NaHS treatment reversed these changes in aortic tissue, indicating that H_2_S can inhibit osteogenic transition in aorta of rats with DN.

### NaHS treatment rescues the elastin level in aortic tissue of rats with DN

**Fig. 6:**
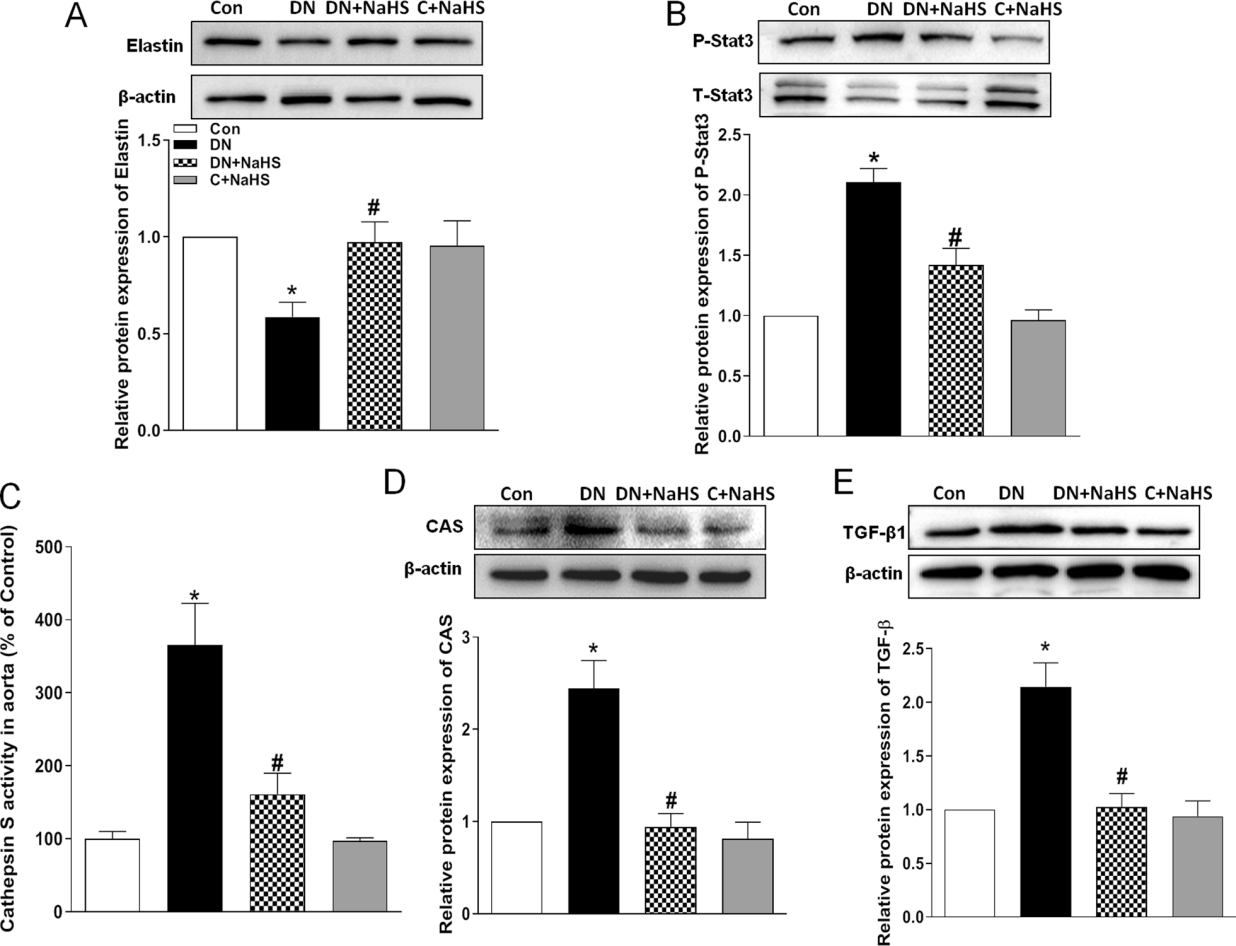
NaHS reduced aortic CAS activity and its protein expression, Stat3 phosphorylation, TGF-β1 protein level and up-regulated elastin protein level in DN rats. Western blot method was used to examine the protein expressions of Stat3, CAS, TGF-β1 and elastin in DN rats after 8 weeks of NaHS (50 μmol/kg/day) treatment. β-actin or total Stat3 from the same blot was control for protein loading. CAS: cathepsin S. C: control. Results are from one representative experiment and relative to total Stat3 or β-actin expression. n = 6 ~ 8 rats (**C**) and n = 3 ~ 5 rats (**A**, **B**, **D**, **E**). Values represent the means ± SEM. *p < 0.05 compared to control rats. #*p* < 0.05 compared to DN rats

In vitro study demonstrates that H_2_S prevents elastin loss and attenuates calcification induced by high levels of glucose and phosphate in VSMCs through suppression of Stat3/Cathepsin S Signaling Pathway [13]. To further confirm the possible mechanisms in aorta of rats with DN, we determined the activities of Stat3 and CAS. As shown in Fig. 6, both activities of Stat3 (Fig. 6B) and CAS (Fig. 6C) were elevated in the arterial wall of DN rats, whereas Western blots demonstrated that the protein expression of elastin was reduced, and the CAS protein expression was increased. The elevated CAS activity may be from the upregulated CAS protein expression, and NaHS treatment abolished the upregulated Stat3 and CAS activity/protein expression in aortic tissue of DN rats (Fig. 6B, C, D). CAS plays an important role in elastin degradation, and its activity regulates elastin level in aortic wall [34]. As expected, NaHS treatment also improved the reduced elastin level in the DN aorta (Fig. 6A). Stat3 activation can also increase TGF-β1 signaling that promotes the calcification of VSMCs [31, 32]. In this experiment, NaHS treatment indeed reduced the TGF-β1 protein expression in aorta of DN rats. Our findings suggest that NaHS treatment may rescue the reduced elastin level through the reduction of the Stat3 activation, CAS activity and TGF-β1 level.

## Discussion

The present study revealed three novel findings. First, H_2_S effectively attenuated AMC in rats with DN. Second, NaHS treatment not only suppressed the phenotype switching, but also up-regulated the elastin level in the aorta. Third, the recovery of elastin level may be due to the reduction of Stat3 activation, CAS activity and TGF-β1 level in the presence of H_2_S. These results indicate that H_2_S suppresses the osteogenic phenotype switching of VSMCs and retards the progression of AMC in DN. The protective effect of H_2_S may be related to the upregulation of elastin level through the decreases in Stat3 activation, CAS activity and TGF-β1 level.

It has been reported that high glucose concentration aggravates high phosphate-induced osteogenic phenotype switching and calcification in cultured VSMCs [35, 36]. Moreover, previous study demonstrated that H_2_S attenuates calcification induced by high glucose in smooth muscle cells under calcifying medium containing high levels of β-glycerophosphate (β-GP) [13]. In the present study, we confirmed that DN model created with a high-fat diet (45% kcal as fat) containing 0.75% adenine, and a single intraperitoneal injection of STZ (ip, 35 mg/kg; Sigma) showed high levels of blood glucose and blood phosphorus in plasma. Furthermore, we found that H_2_S administration not only inhibited the phenotype transformation of medial layer of aorta, but also ameliorated AMC in rats with DN.

The occurrence of VC may be associated with the reductions of CSE expression in aorta and H_2_S levels in blood of DN rats, suggests a causal role for endogenous H_2_S effects on calcification. In previous study, we found that pharmacologic inhibition of CSE activity aggravated calcification, promoted the further activation of Stat3, increased CAS activity, and reduced the elastin expression in smooth muscle cells (SMCs) cultured by high levels of glucose and phosphate (HGP) [13]. In the present study, exogenous H_2_S administration reduced the blood glucose, creatinine and urea nitrogen levels, ameliorated VC, inhibited the activation of Stat3, reduced CAS activity, decreased TGF-β1 level and improved the elastin expression in vessel wall of aorta in rats with DN. All of these results suggest that endogenous H_2_S may have the inhibitory effects on VC, probably via inhibiting Stat3 activation, CAS activity and TGF-β1 protein expression. The improvement of glycemic control and renal function provided by H_2_S may be also involved in the inhibition of VC in rats with DN.

Stat3, an inducible monomeric transcription factor, could be activated by high glucose to induce ER stress, inflammation and oxidative stress in diabetic retinopathy or diabetic nephropathy [37–40]. In this study, Stat3 was activated in tunica media of aorta in DN rats, and it was suppressed by H_2_S administration. Previous study has been demonstrated that suppression of Stat3 or knockdown of Stat3 mRNA displayed inhibitory effect on calcification of VSMCs [13]. So our data indicate that Stat3 activation may involve the regulation of VC in rats with DN, which can be inhibited by H_2_S administration. IL-6/JAK can activate STAT3 pathway or receptors with intrinsic kinase activity such as EGFR and VEGFR directly or indirectly induce STAT3 activation in some cells [41]. In this study, whether intrinsic kinase activity involving H_2_S- regulated Stat3 activation needs to be further explored.

Elastin is synthesized and secreted from VSMCs for the maintenance of the vascular environment and the VSMC phenotype [27, 28]. Aortic stiffening and remodelling were related to elastin loss or disruption of elastin fibres in diabetic rats [42] or CKD [30]. CAS can degrade the basement membrane and surrounding extracellular matrix of arterial walls that participates in regulating elastin degradation and calcification [43]. In this study, H_2_S inhibited the increases of CAS activity and its protein expression in aorta of DN rats. Interestingly, we observed that elastin was reduced markedly in aorta of rats with DN, and they were improved by NaHS treatment. STAT3 can mediate the increase of CAS activity in dendritic cells [44]. Moreover, H_2_S can mediate neuroinflammation and Aβ1-42 production by suppressing the activation of STAT3 and CAS [45]. Our previous study also showed that H_2_S inhibited the increases of CAS expression and activity in vitro, and these effects were markedly inhibited by the Stat3 inhibitor. Moreover, down-regulation of Stat3 expression also inhibited CAS expression [13]. So H_2_S inhibits calcification in this study may be partly associated with the inhibition of Stat3/CAS pathway for increasing elastin level in aorta. Therefore, the knockdown of CAS expression in the tunica media may be a better strategy for treatment of AMC in patient with DN. In addition, H_2_S can inhibit the activation of Stat3 to attenuate TGF-β1 signaling involving the improvement of liver fibrosis [31]. Moreover, TGF-β1 synergistically amplified responses with elastin degradation products involving VC [33]. So it is possible that H_2_S can inhibit the activation of Stat3 to attenuate TGF-β1 effects for inhibiting VC. Indeed, we found that TGF-β1 protein expression was effectively inhibited by H_2_S. This result suggests that the inhibition of Stat3-TGF-β1 pathway may be another mechanism of H_2_S in improving AMC in DN state.

In conclusion, this study demonstrated that H_2_S inhibits VC in rats with DN that further confirmed the previous results of in vitro studies [13]. H_2_S may have a clinical significance for treating AMC in people with DN by decreasing Stat3 activation, CAS activity and TGF-β1 level for increasing local elastin level. Further in vivo studies are needed to determine the exact mechanism by which H_2_S affects elastin level, Stat3 activation, CAS activity and TGF-β1 level involving the inhibition of VC.

## Supplementary Information

**Additional file 1**. The original and unclipped Western blotting images.

**Additional file 2**. ARRIVE guidelines.

## Data Availability

The datasets used and/or analyzed in this study will be made available by the authors on reasonable request.

## References

[1] Chistiakov DA, Sobenin IA, Orekhov AN, Bobryshev YV (2014). Mechanisms of medial arterial calcification in diabetes. Curr Pharm Des.

[2] Lioufas NM, Pedagogos E, Hawley CM, Pascoe EM, Elder GJ, Badve SV, Valks A, Toussaint ND (2020). Aortic calcification and arterial stiffness burden in a chronic kidney disease cohort with high cardiovascular risk: baseline characteristics of the impact of phosphate reduction on vascular end-points in chronic kidney disease trial. Am J Nephrol.

[3] Ho CY, Shanahan CM (2016). Medial arterial calcification: an overlooked player in peripheral arterial disease. Arterioscler Thromb Vasc Biol.

[4] Jeffcoate WJ, Rasmussen LM, Hofbauer LC, Game FL (2009). Medial arterial calcification in diabetes and its relationship to neuropathy. Diabetologia.

[5] Hruska KA, Mathew S, Lund R, Qiu P, Pratt R (2008). Hyperphosphatemia of chronic kidney disease. Kidney Int.

[6] Disthabanchong S, Srisuwarn P (2019). Mechanims of vascular calcification in kidney dease. Adv Chronic Kidney Dis.

[7] Klimentová A, Ságová I, Prídavková D, Kantárová D, Makovický P, Sadloňová J, Mokáň M (2016). Diabetic kidney disease 3rd stage - laboratory markers of mineral bone disorder. Vnitr Lek.

[8] Baktiroglu S, Yanar F, Ozata IH, Oner G, Ercan D (2016). Arterial disease and vascular access in diabetic patients. J Vasc Access.

[9] Georgiadis GS, Argyriou C, Antoniou GA, Kantartzi K, Kriki P, Theodoridis M, Thodis E, Lazarides MK (2015). Upper limb vascular calcification score as a predictor of mortality in diabetic hemodialysis patients. J Vasc Surg.

[10] Schinzari F, Tesauro M, Bertoli A, Valentini A, Veneziani A, Campia U, Cardillo C (2019). Calcification biomarkers and vascular dysfunction in obesity and type 2 diabetes: influence of oral hypoglycemic agents. Am J Physiol Endocrinol Metab.

[11] Durham AL, Speer MY, Scatena M, Giachelli CM, Shanahan CM (2018). Role of smooth muscle cells in vascular calcification: implications in atherosclerosis and arterial stiffness. Cardiovasc Res.

[12] Kong Y, Liang Q, Chen Y, Yang P, Liu X, Li Y, Feng S, Wu J, Liu W, Tang J, Yu H, Ou JS, Lu L, Yan J (2018). Hyaluronan negatively regulates vascular calcification involving BMP2 signaling. Lab Investig.

[13] Zhou YB, Zhou H, Li L, Kang Y, Cao X, Wu ZY, Ding L, Sethi G, Bian JS (2019). Hydrogen sulfide prevents elastin loss and attenuates calcification induced by high glucose in smooth muscle cells through suppression of Stat3/Cathepsin S signaling pathway. Int J Mol Sci.

[14] Liu YH, Lu M, Hu LF, Wong PT, Webb GD, Bian JS (2012). Hydrogen sulfide in the mammalian cardiovascular system. Antioxid Redox Signal.

[15] Liu YH, Lu M, Xie ZZ, Hua F, Xie L, Gao JH, Koh YH, Bian JS (2014). Hydrogen sulfide prevents heart failure development via inhibition of renin release from mast cells in isoproterenol-treated rats. Antioxid Redox Signal.

[16] Cao X, Zhang W, Moore PK, Bian J (2019). Protective smell of hydrogen sulfide and polysulfide in cisplatin-induced nephrotoxicity. Int J Mol Sci.

[17] Yang G, Wang R (2015). H_2_S and blood vessels: an overview. Handb Exp Pharmacol.

[18] El-Sayed SS, Zakaria MN, Abdel-Ghany RH, Abdel-Rahman AA (2016). Cystathionine-gamma lyase-derived hydrogen sulfide mediates the cardiovascular protective effects of moxonidine in diabetic rats. Eur J Pharmacol.

[19] Zhong X, Wang Y, Wu J, Sun A, Yang F, Zheng D, Li T, Dong S, Zhao Y, Yang G, Xu C, Sun D, Lu F, Zhang W (2015). Calcium sensing receptor regulating smooth muscle cells proliferation through initiating cystathionine-gamma-lyase/hydrogen sulfide pathway in diabetic rat. Cell Physiol Biochem.

[20] Li F, Luo J, Wu Z, Xiao T, Zeng O, Li L, Li Y, Yang J (2016). Hydrogen sulfide exhibits cardioprotective effects by decreasing endoplasmic reticulum stress in a diabetic cardiomyopathy rat model. Mol Med Rep.

[21] Bos EM, Leuvenink HG, Snijder PM, Kloosterhuis NJ, Hillebrands JL, Leemans JC, Florquin S, van Goor H (2009). Hydrogen sulfide-induced hypometabolism prevents renal ischemia/reperfusion injury. J Am Soc Nephrol.

[22] Yamamoto J, Sato W, Kosugi T, Yamamoto T, Kimura T, Taniguchi S, Kojima H, Maruyama S, Imai E, Matsuo S, Yuzawa Y, Niki I (2013). Distribution of hydrogen sulfide (H(2)S)-producing enzymes and the roles of the H(2)S donor sodium hydrosulfide in diabetic nephropathy. Clin Exp Nephrol.

[23] Aghagolzadeh P, Radpour R, Bachtler M, van Goor H, Smith ER, Lister A, Odermatt A, Feelisch M, Pasch A (2017). Hydrogen sulfide attenuates calcification of vascular smooth muscle cells via KEAP1/NRF2/NQO1 activation. Atherosclerosis.

[24] Shrivastava K, Llovera G, Recasens M, Chertoff M, Giménez-Llort L, Gonzalez B, Acarin L (2013). Temporal expression of cytokines and signal transducer and activator of transcription factor 3 activation after neonatal hypoxia/ischemia in mice. Dev Neurosci.

[25] Demyanets S, Kaun C, Rychli K, Pfaffenberger S, Kastl SP, Hohensinner PJ, Rega G, Katsaros KM, Afonyushkin T, Bochkov VN, Paireder M, Huk I, Maurer G, Huber K, Wojta J (2011). Oncostatin M-enhanced vascular endothelial growth factor expression in human vascular smooth muscle cells involves PI3K-, p38 MAPK-, Erk1/2- and STAT1/STAT3-dependent pathways and is attenuated by interferon-gamma. Basic Res Cardiol.

[26] Johnson AW, Kinzenbaw DA, Modrick ML, Faraci FM (2013). Small-molecule inhibitors of signal transducer and activator of transcription 3 protect against angiotensin II-induced vascular dysfunction and hypertension. Hypertension.

[27] Simpson CL, Lindley S, Eisenberg C, Basalyga DM, Starcher BC, Simionescu DT, Vyavahare NR (2007). Toward cell therapy for vascular calcification: osteoclast-mediated demineralization of calcified elastin. Cardiovasc Pathol.

[28] Vrhovski B, Weiss AS (1998). Biochemistry of tropoelastin. Eur J Biochem.

[29] Kakutani Y, Shioi A, Shoji T, Okazaki H, Koyama H, Emoto M, Inaba M (2015). Oncostatin M Promotes Osteoblastic differentiation of human vascular smooth muscle cells through JAK3-STAT3 pathway. J Cell Biochem.

[30] Aikawa E, Aikawa M, Libby P, Figueiredo JL, Rusanescu G, Iwamoto Y, Fukuda D, Kohler RH, Shi GP, Jaffer FA, Weissleder R (2009). Arterial and aortic valve calcification abolished by elastolytic cathepsin S deficiency in chronic renal disease. Circulation.

[31] Gong Z, Ye H, Huo Y, Wang L, Huang Y, Huang M, Yuan X (2018). S-allyl-cysteine attenuates carbon tetrachloride-induced liver fibrosis in rats by targeting STAT3/SMAD3 pathway. Am J Transl Res.

[32] He F, Li L, Li PP, Deng Y, Yang YY, Deng YX, Luo HH, Yao XT, Su YX, Gan H, He BC (2020). Cyclooxygenase-2/sclerostin mediates TGF-beta1-induced calcification in vascular smooth muscle cells and rats undergoing renal failure. Aging (Albany NY).

[33] Simionescu A, Philips K, Vyavahare N (2005). Elastin-derived peptides and TGF-beta1 induce osteogenic responses in smooth muscle cells. Biochem Biophys Res Commun.

[34] Swaroop M, Bradley K, Ohura T, Tahara T, Roper MD, Rosenberg LE, Kraus JP (1992). Rat cystathionine beta-synthase. Gene organization and alternative splicing. J Biol Chem.

[35] Merjanian R, Budoff M, Adler S, Berman N, Mehrotra R (2003). Coronary artery, aortic wall, and valvular calcification in nondialyzed individuals with type 2 diabetes and renal disease. Kidney Int.

[36] Chen NX, Duan D, O'Neill KD, Moe SM (2006). High glucose increases the expression of Cbfa1 and BMP-2 and enhances the calcification of vascular smooth muscle cells. Nephrol Dial Transplant.

[37] Darnell JE, Kerr IM, Stark GR (1994). Jak-STAT pathways and transcriptional activation in response to IFNs and other extracellular signaling proteins. Science.

[38] Kumar A, Commane M, Flickinger TW, Horvath CM, Stark GR (1997). STATs and gene regulation. Science.

[39] Chen Y, Wang JJ, Li J, Hosoya KI, Ratan R, Townes T, Zhang SX (2012). Activating transcription factor 4 mediates hyperglycaemia-induced endothelial inflammation and retinal vascular leakage through activation of STAT3 in a mouse model of type 1 diabetes. Diabetologia.

[40] Li L, Shaw PE (2004). A STAT3 dimer formed by inter-chain disulphide bridging during oxidative stress. Biochem Biophys Res Commun.

[41] Ibrahim SA, Gadalla R, El-Ghonaimy EA, Samir O, Mohamed HT, Hassan H, Greve B, El-Shinawi M, Mohamed MM, Götte M (2017). Syndecan-1 is a novel molecular marker for triple negative inflammatory breast cancer and modulates the cancer stem cell phenotype via the IL-6/STAT3, Notch and EGFR signaling pathways. Mol Cancer.

[42] Li WB, Zhao J, Liu L, Wang ZH, Han L, Zhong M, Zhang Y, Zhang W, Tang MX (2015). Silencing of activin receptor-like kinase 7 alleviates aortic stiffness in type 2 diabetic rats. Acta Diabetol.

[43] Figueiredo JL, Aikawa M, Zheng C, Aaron J, Lax L, Libby P, de Lima Filho JL, Gruener S, Fingerle J, Haap W, Hartmann G, Aikawa E (2015). Selective cathepsin S inhibition attenuates atherosclerosis in apolipoprotein E-deficient mice with chronic renal disease. Am J Pathol.

[44] Kitamura H, Kamon H, Sawa S, Park SJ, Katunuma N, Ishihara K, Murakami M, Hirano T (2005). IL-6-STAT3 controls intracellular MHC class II alphabeta dimer level through cathepsin S activity in dendritic cells. Immunity.

[45] Cao L, Cao X, Zhou Y, Nagpure BV, Wu ZY, Hu LF, Yang Y, Sethi G, Moore PK, Bian JS (2018). Hydrogen sulfide inhibits ATP-induced neuroinflammation and Abeta(1–42) synthesis by suppressing the activation of STAT3 and cathepsin S. Brain Behav Immun.

