# Supplementary material for: Hydrogen sulfide prevents arterial medial calcification in rats with diabetic nephropathy
Source: BMC Cardiovasc Disord. 2021 Oct 13;21:495. doi: 10.1186/s12872-021-02307-9 (PMC8515673; doi:10.1186/s12872-021-02307-9)
Supplement: Supplementary file 1 — Additional file 1. The original and unclipped Western blotting images. [file 12872_2021_2307_MOESM1_ESM.pdf]

ONLINE SUPPLEMENT

Hydrogen sulfide prevents arterial medial calcification in rats with diabetic nephropathy

Fang-Zheng Wang[1, a], Hong Zhou [1, a], Hong-Yu Wang[1], Hang-Bing Dai[1], Qing Gao[1], Pei Qian[1], Ye-Bo Zhou[1, *]

[1]Department of Physiology, Nanjing Medical University, Nanjing, Jiangsu 210029, China.

Running title: Hydrogen sulfide prevents calcification

[a]The two authors contributed equally to this work.

*****Correspondence to:

Dr. Ye-Bo Zhou, M.D., Ph.D.

Department of **Physiology**, Nanjing Medical University 101 Longmian Road, Nanjing 211166, China Tel: +86-25-86862885

Fax: +86-25-86862885

Email: zhouyebo666@njmu.edu.cn

Supplemental Figure.

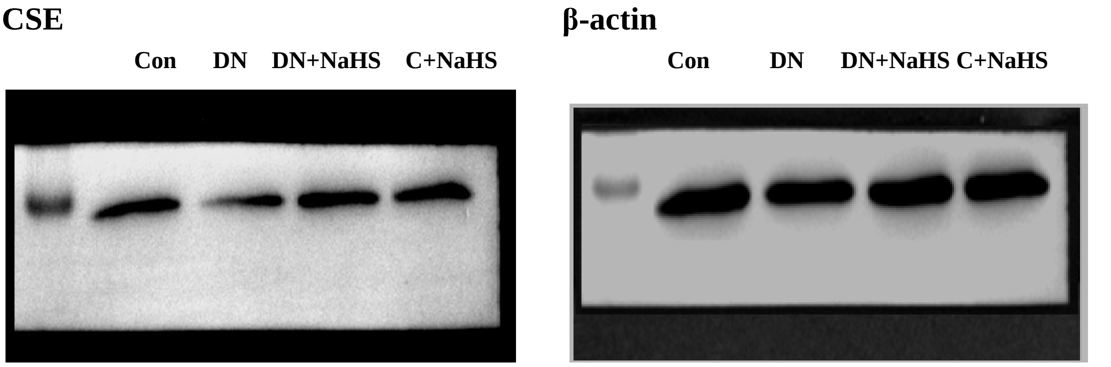

Figure S8. The CSE (44 kD) was cut from the whole membrane according to the protein molecular weight indicated by the markers. The same membrane was used again for getting the β-actin (42 kD) blot after removal of the primary antibody by using stripping buffer. The two blots as representative images were used for the Figure 5B in the manuscript file.

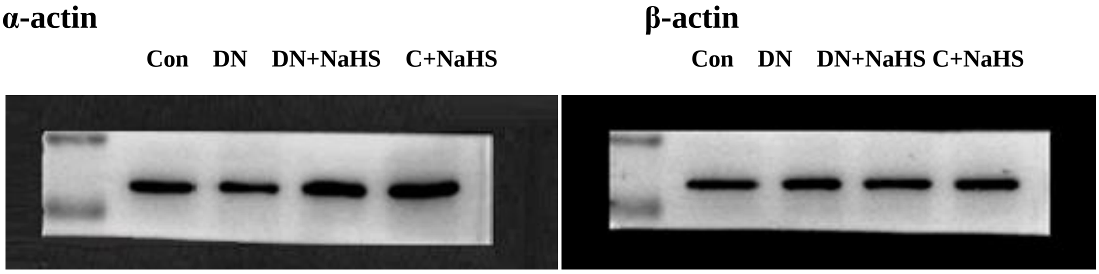

Figure S2. The membrane about the α-actin (42 kD) blot was cut from the whole membrane according to the protein molecular weight indicated by the markers. The same membrane was used again for getting the β-actin (42 kD) blot after removal of the primary antibody α-actin by using stripping buffer. The two blots as representative images were used for the Figure 5C in the manuscript file.

SM-22α **β-actin**

Con DN DN+NaHS C+NaHS **Con DN DN+NaHS C+NaHS**

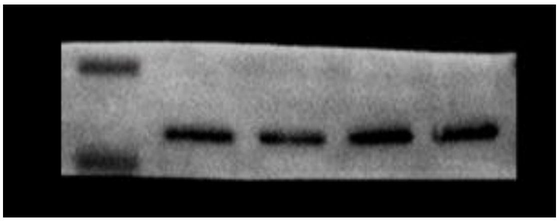

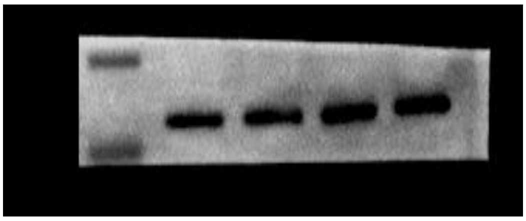

Figure S3. These two membranes about SM-22α (22 kD) and β-actin (42 kD) were cut from the same whole membrane according to the protein molecular weight indicated by the markers. The two blots as representative images were used for the Figure 5D in the manuscript file.

Cbfα-1 **β-actin**

Con DN DN+NaHS C+NaHS **Con DN DN+NaHS C+NaHS**

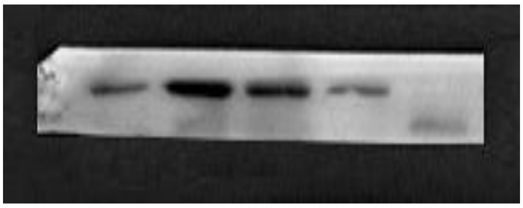

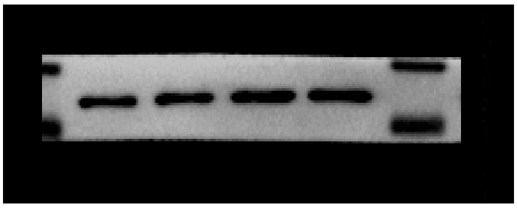

Figure S4. These two membranes about Cbfα-1 (55 kD) and β-actin (42 kD) were cut from the same whole membrame ccording to the protein molecular weight indicated by the markers. The two blots as representative images were used for the Figure 5E in the manuscript file.

Elastin **β-actin**

Con DN DN+NaHS C+NaHS **Con DN DN+NaHS C+NaHS**

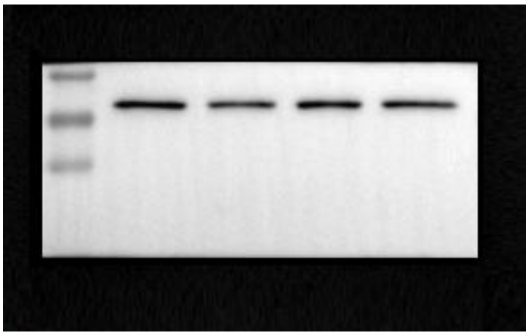

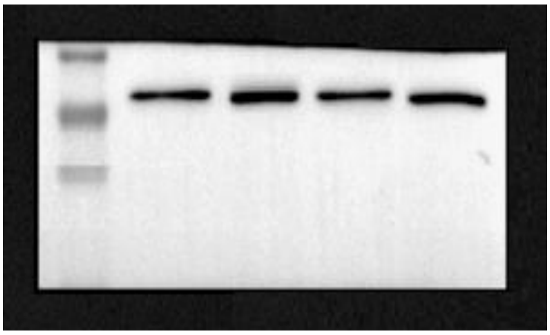

Figure S5. These two membranes about elastin (70 kD) and β-actin (42 kD) were cut from the same whole membrane according to the protein molecular weight indicated by the markers. The two blots as representative images were used for the Figure 6A in the manuscript file.

P-stat3 **P-stat3**

Con DN DN+NaHS C+NaHS Con DN DN+NaHS C+NaHS

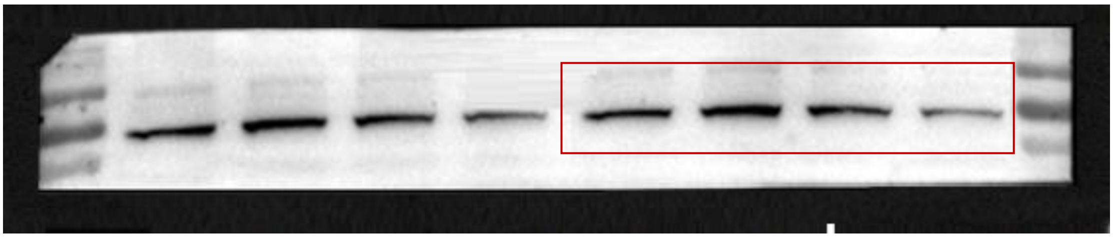

T-stat3

Con DN DN+NaHS C+NaHS Con DN DN+NaHS C+NaHS

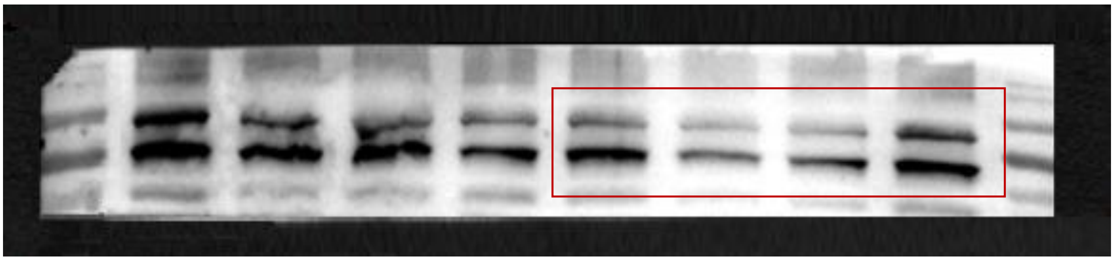

Figure S6. The membrane about the P-stat3 (80 kD) blot were cut from the whole membrane according to the protein molecular weight indicated by the markers. The same membrane was used again for getting the T-stat3 (80 kD) blot after removal of the primary antibody P-stat3 by using stripping buffer. The marked in red blots as representative images were used for the Figure 6B in the manuscript file.

CAS **β-actin**

Con DN DN+NaHS C+NaHS Con DN DN+NaHS C+NaHS

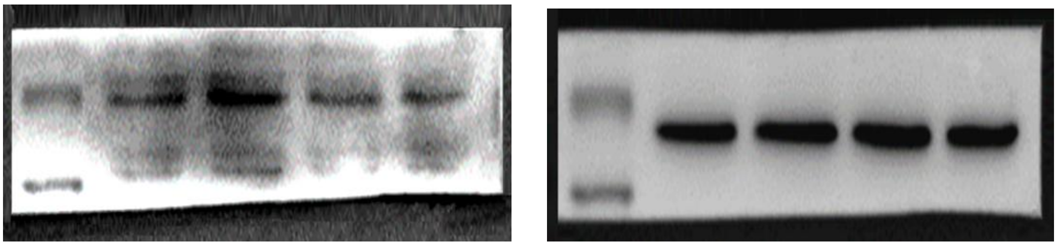

Figure S7. The CAS (37 kD) was cut from the whole membrane according to the protein molecular weight indicated by the markers. The same membrane was used again for getting the β-actin (42 kD) blot after removal of the primary antibody by using stripping buffer. The two blots as representative images were used for the Figure 6D in the manuscript file.

TGF-β1 **β-actin**

Con DN DN+NaHS C+NaHS Con DN DN+NaHS C+NaHS

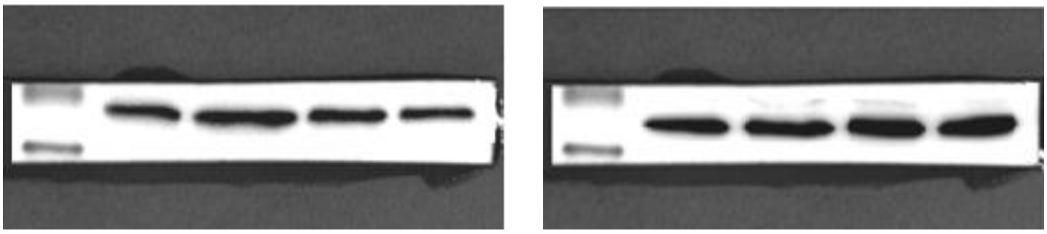

Figure S8. The TGF-β1 (44 kD) was cut from the whole membrane according to the protein molecular weight indicated by the markers. The same membrane was used again for getting the β-actin (42 kD) blot after removal of the primary antibody by using stripping buffer. The two blots as representative images were used for the Figure 6E in the manuscript file.
